# Penehyclidine hydrochloride for treating postoperative nausea and vomiting after laparoscopic bariatric surgery: a double-blinded randomized controlled trial

**DOI:** 10.1186/s12871-023-02078-0

**Published:** 2023-04-24

**Authors:** Xiahao Ding, Dapeng Chen, Jinxing Che, Siyang Xu, Hui Liang, Bo Gui

**Affiliations:** 1 Department of Anesthesiology and Perioperative Medicine, 1st Affiliated Hospital with Nanjing Medical University, Nanjing, 210029 China; 2 Department of Anesthesiology, The Huai’an Maternity and Child Healthcare Hospital, Huai’an, 223002 China; 3 Department of Anesthesiology, Jiangsu Province Official Hospital, Nanjing, 210009 China; 4 Department of General Surgery, 1st Affiliated Hospital with Nanjing Medical University, Nanjing, 210029 China

**Keywords:** Laparoscopic bariatric surgery, Penehyclidine hydrochloride, Postoperative nausea and vomiting, Time to first flatus

## Abstract

**Background:**

Postoperative nausea and vomiting (PONV) is a common and distressing complication of laparoscopic bariatric surgery (LBS). Penehyclidine hydrochloride has been reported to be effective in preventing PONV. Considering the potential preventive effects of penehyclidine against PONV, we hypothesized that intravenous infusion of penehyclidine may alleviate PONV within the first 48 h in patients scheduled for LBS.

**Methods:**

Patients who underwent LBS were randomly assigned (1:2) to receive saline (Control group, *n* = 113) or a single intravenous dose of penehyclidine 0.5 mg (PHC group, *n* = 221). The primary outcome was incidence of PONV within the first 48 h postoperatively. Secondary endpoints included severity of PONV, need for rescue antiemetic therapy, volume of water intake, and time to first flatus.

**Results:**

PONV occurred in 159 (48%) patients within the first 48 h postoperatively, including 51% in the Control group and 46% in the PHC group. There was no significant difference in the incidence or severity of PONV between the two groups (*P* > 0.05). Within the first 24 h and 24–48 h, no significant difference was found in incidence or severity of PONV, postoperative nausea, postoperative vomiting, need for rescue antiemetic therapy, or volume of water intake (*P* > 0.05). Kaplan–Meier curves showed that penehyclidine was significantly associated with a prolonged time to first flatus (median onset time: 22 h vs. 21 h, *P* = 0.036).

**Conclusions:**

Penehyclidine did not decrease incidence and severity of PONV in patients undergoing LBS. However, a single intravenous dose of penehyclidine (0.5 mg) was associated with a slightly prolonged time to first flatus.

**Trial registration:**

Chinese Clinical Trial Registry (ChiCTR2100052418, http://www.chictr.org.cn/showprojen.aspx?proj=134893, date of registration: 25/10/2021).

## Background

Laparoscopic bariatric surgery (LBS) has been generally recognized as a safe and effective procedure for obese adults, given the global prevalence of overweight and obesity [1, 2]. Postoperative nausea and vomiting (PONV) is a common and distressing complication after a surgery involving general anesthesia. PONV occurs frequently after LBS, at an incidence of 35–89% [3, 4]. Moreover, PONV has been shown to be strongly correlated with several adverse clinical consequences, such as patient dissatisfaction, incision disruption, water-electrolyte imbalance, prolonged length of hospital stay, and increased medical expenses [3, 5]. As such, increasing attention has been paid to this common and burdensome complaint. Multiple intervention strategies are required to prevent and manage PONV after LBS.

The etiology of PONV is multifactorial involving patient, surgical, and anesthesia-related factors. Several types of neurotransmitters, including serotonin, dopamine, muscarine, neurokinin-1, opioids, and histamine are closely associated with PONV [6]. Stimulation of vestibular-cochlear, glossopharyngeal, and vagus nerves may also be responsible for occurrence of PONV. Preclinical and clinical evidence revealed that multi-modal approach to target these pharmacological sites is important for controlling PONV [7]. Penehyclidine hydrochloride is an anticholinergic drug widely used for the treatment of organic phosphorus poisoning, as preanesthetic medication, and for the protection of certain visceral organs [8]. Prophylactic medication with penehyclidine may prevent PONV in patients undergoing gynecological laparoscopic surgery, bimaxillary orthognathic surgery, and strabismus surgery [9–11]. The underlying mechanism of penehyclidine may be related to the inactivation of the M3 muscarinic acetylcholine receptor [12, 13]. A combination of prophylactic antiemetic drugs with different mechanisms of action should be administered to patients with moderate to high risk of developing PONV.

Considering the potential preventive effects of penehyclidine against PONV, we hypothesized that intravenous infusion of penehyclidine may alleviate PONV and improve postoperative recovery within the first 48 h in patients scheduled for LBS.

## Methods

### Study design and ethical approval

This prospective, double-blinded, randomized controlled trial was conducted in an accredited bariatric center of a tertiary hospital in China between 01/11/2021 and 13/05/2022. The study protocol was approved by the Ethics Committee (No. 2020-SR-059, 11/03/2020) and registered in the Chinese Clinical Trial Registry (ChiCTR2100052418, 25/10/2021). All participants signed an informed consent form before enrollment in the study. This study is reported in line with the Consolidated Standards of Reporting Trials (CONSORT) guidelines.

### Study participants

The same surgeon performed all procedures, including laparoscopic sleeve gastrectomy (LSG), laparoscopic sleeve gastrectomy plus duodenojejunal bypass (LSG-DJB), laparoscopic sleeve gastrectomy plus jejunojejunal bypass (LSG-JJB), and one anastomosis gastric bypass (OAGB). The inclusion criteria were the American Society of Anesthesiologists (ASA) physical status I–III, aged 18–60 years, and scheduled for elective LBS under general anesthesia. The exclusion criteria were listed as followed: contraindication to penehyclidine administration (glaucoma, basal ganglia disease, Parkinson’s disease, pheochromocytoma, myasthenia gravis, severe central nervous depression, and ECG showing prolonged Q-T interval) and already using antiemetic drugs within 48 h before surgery.

### Randomization and blinding

The participants were randomly assigned (1:2) using a computer-generated randomization system to receive saline (Control group) or a single intravenous dose of penehyclidine 0.5 mg (PHC group) (Chengdu Lisite Pharmaceutical Co., Ltd., Chengdu, China) after enrollment into the trial. The random allocation sequence was sealed in opaque envelopes. Participants, care providers, and investigators were all blinded to treatment allocation. For safety reasons, the anesthesiologists responsible for administering the anesthetic were aware of the grouping. However, they were not responsible for the postoperative assessment and data collection.

### Anesthesia protocol

After the patients arrived in the operating room, electrocardiogram (ECG), blood pressure (BP), oxygen saturation (SpO_2_), heart rate (HR), end-tidal carbon dioxide (EtCO_2_), and body temperature (T) were monitored. All patients received a loading infusion of lactated Ringer’s solution (10 ml/kg). Anesthesia induction was accomplished using dexamethasone 10 mg, midazolam 0.05 mg/kg, propofol 1.5‒2.5 mg/kg, fentanyl 4–6 µg/kg, plus rocuronium 0.9 mg/kg or cis-atracurium 0.15 mg/kg to facilitate tracheal intubation. Meanwhile, patients in the PHC group received a single intravenous dose of penehyclidine 0.5 mg, while patients in the Control group received same volume of saline. All patients were endotracheally intubated and mechanically ventilated with a tidal volume of 6–8 ml/kg (ideal body weight) and a respiratory rate of 12–16 breaths per minute to maintain EtCO_2_ at the level of 35–45 mmHg. Anesthesia was maintained with a continuous infusion of propofol 100–200 µg/kg/min, remifentanil 0.05–0.15 µg/kg/min, rocuronium 5–10 µg/kg/min or cis-atracurium 1–3 µg/kg/min. The depth of anesthesia was monitored using the bispectral index, which was maintained between 40 and 60. Neuromuscular blocking drugs (NMBDs) were selected according to the choice of reversal agent. Only patients with a BMI ≥ 35 kg/m^2^ undergoing elective LBS are recommended to receive sugammadex for neuromuscular blockade (NMB) reversal in our center. If NMB was reversed by sugammadex, rocuronium would be used for both induction and maintenance. If neostigmine, cis-atracurium would be used instead. The sevoflurane concentration was adjusted as necessary. BP was maintained at a fluctuation of ± 20% of the baseline value by adjusting the depth of anesthesia or using vasoactive agents during surgery. All patients received a single intravenous dose of palonosetron (0.25 mg) 30 min before the completion of surgery. Continuous infusion of rocuronium or cis-atracurium was stopped after deflation of the pneumoperitoneum, and the infusion of propofol and remifentanil was discontinued at completion of surgery. Patients were admitted to the post-anesthesia care unit (PACU) with an endotracheal tube for recovery from anesthesia. We used clinically physical signs for assessing the recovery of neuromuscular function and guiding the use of antagonists of NMBDs. NMB was allowed to recover spontaneously to the moderate block state, and the residual effects of rocuronium were antagonized via administration of either sugammadex 200 mg or neostigmine 2 mg plus atropine 1 mg. Ventilatory support was maintained until a unified extubation standard was achieved. Tracheal extubation was performed according to a standardized protocol: fully conscious, stable circulation (BP and HR remain within 20% of the baseline values without any inotropes), a respiratory rate < 30 breaths/min, maximal inspiratory pressure < − 20 cmH_2_O, tidal volume > 6 ml/kg, SpO_2_ > 93% as well as ability to lift head for 5 s. The criterion used for patient discharge from PACU was the achievement of a modified Aldrete score ≥ 9 [14]. After patients returned to the ward, an intramuscular injection of metoclopramide 10 mg was administered as an initial rescue antiemetic therapy at the request of a patient or when a patient experienced > 5 episodes of vomiting within 24 h. If the symptoms persisted one hour after metoclopramide administration, another 10 mg of metoclopramide was administered as a second rescue drug. No more than 20 mg of metoclopramide was allowed in any 24-h period. A total of 10 mg of dezocine was administered intravenously as rescue analgesia in the ward when the visual analog scale score was ≥ 4.

### Data collection

Data on clinical characteristics, including age, sex, body mass index, coexisting disorders, and potential risk factors for PONV, were collected to assess comparability. We also recorded the simplified Apfel score for each patient [15]. The primary outcome was incidence of PONV within the first 48 h postoperatively. Secondary endpoints included severity of PONV, need for rescue antiemetic therapy, volume of water intake, and time to first flatus. PONV was defined as at least one episode of nausea, vomiting, retching, or any combination of these symptoms. PONV was evaluated as follows: I = no nausea or vomiting, II = nausea but no vomiting, III = mild to moderate vomiting, and IV = severe and frequent vomiting more than five times within 24 h. The severity of postoperative nausea (PON) was assessed with a numeric rating scale (I = mild, II = moderate, III = severe). The severity of postoperative vomiting (POV) was recorded according to the number of vomiting episodes (I = no vomiting, II = vomiting episodes occurring 1–2 times within 24 h, III = vomiting episodes occurring 3–5 times within 24 h, IV = vomiting episodes occurring > 5 times within 24 h). The simplified Apfel score contains four risk factors, including female gender, history of motion sickness or PONV, nonsmoking status, and postoperative opioid use. Intraoperative opioid consumption means the total amount of opioids used in the operating room and PACU. All opioid doses were converted to morphine intravenous equivalent. According to clinical guidelines, patients were instructed to drink clear liquids for the first 24–48 h after LBS, with the volume gradually increasing to 2 L to promote the recovery of gastrointestinal function [16]. Accordingly, the volume of postoperative water intake was measured during the two periods (0–24 h and 24–48 h). The time to first flatus was defined as the time to the first passage of flatus, as reported by patients, minus the end time of the surgery.

### Sample size

The sample size calculation was based on our preliminary data, which indicated that 55% of the patients experienced PONV within the first 48 h postoperatively. Thus, we considered a 35% reduction in the incidence of PONV as clinically significant. The required sample size was estimated using Power Analysis and Sample Size software (PASS, version 15.0. NCSS, LLC. Kaysville, Utah, USA). The result suggested that 104 patients in the Control group and 207 in the PHC group would be necessary to achieve a power of 90% (*β* = 0.1) with a two-sided confidence interval of 95% (*α* = 0.05). Considering an attrition rate of approximately 10%, we increased the sample size to 346 patients (116 in the Control group and 230 in the PHC group).

### Statistical analysis

Descriptive statistics are presented as mean ± standard deviation for continuous variables and as frequencies or proportions for categorical variables. Normal distribution of the data was confirmed using the Shapiro–Wilk test. For normally distributed data, an independent Student’s *t*-test was used to assess significant differences between the two groups. For data with a skewed distribution, the Mann–Whitney *U* test was used. Categorical variables were analyzed using either the chi-square test or Fisher’s exact test, as appropriate. There were no missing values in our findings. Statistical significance was set at *P* < 0.05. All statistical analyses were performed using IBM SPSS Statistics for Windows (version 21.0. SPSS Inc. Chicago, Illinois, USA).

## Results

**Fig. 1:**
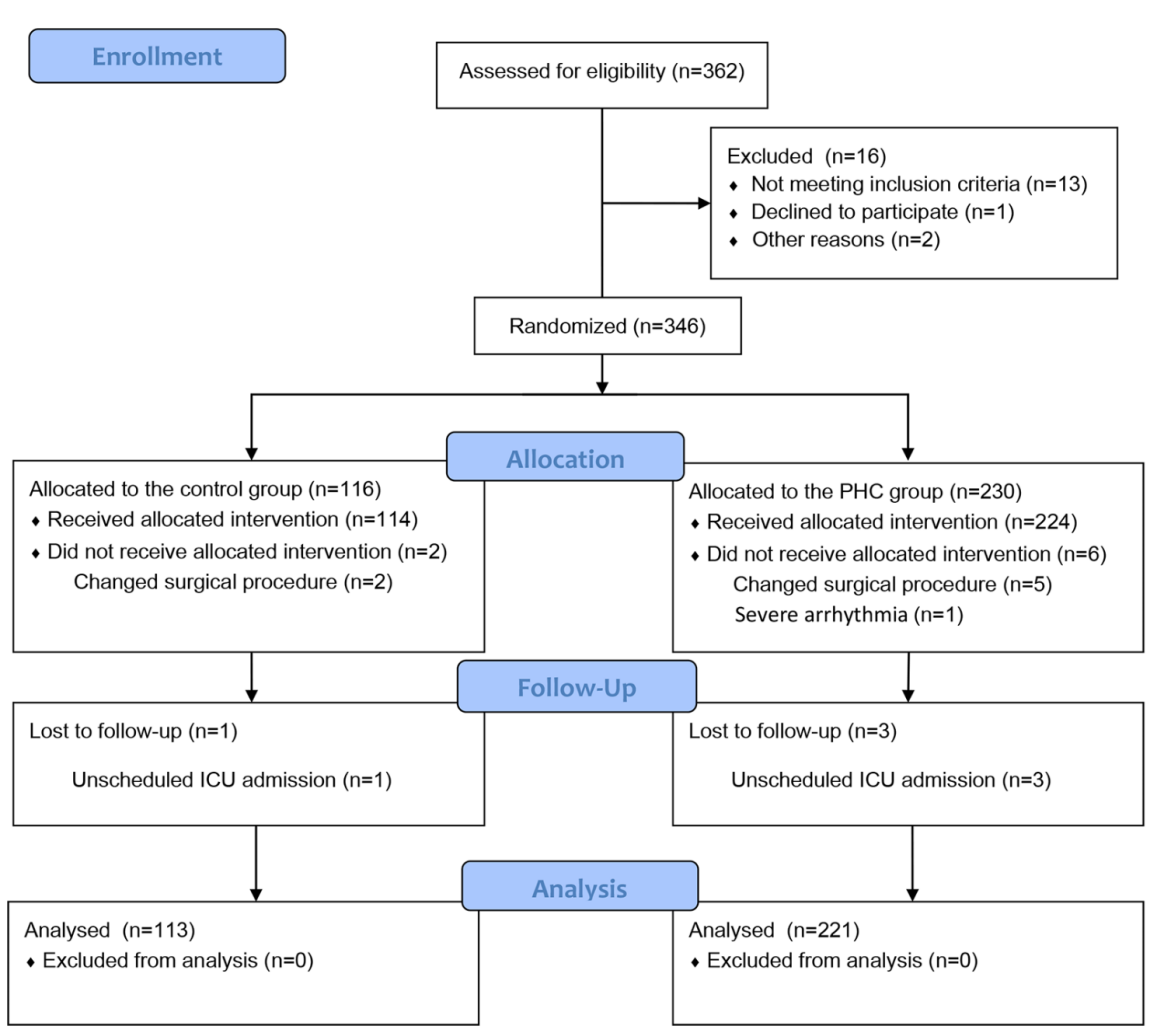
Flow diagram of patients enrolled in the study. *ICU* intensive care units, *PHC* penehyclidine hydrochloride

A flow diagram of the study is shown in Fig. 1. A total of 362 patients undergoing elective LBS were assessed for eligibility, of whom 346 were available for primary analysis between 01/11/2021 and 13/05/2022. Sixteen patients were excluded before randomization because they did not meet the inclusion criteria (*n* = 13), declined to participate (*n* = 1), or case cancellation on the day of surgery (*n* = 2). Three patients in the Control group were excluded because of changes in the surgical procedure during the operation (*n* = 2) and unscheduled ICU admission (*n* = 1). Nine patients in the PHC group were excluded owing to temporary alteration of the surgical procedure (*n* = 5), severe intraoperative arrhythmia (*n* = 1), and unscheduled ICU admission (*n* = 3). Ultimately, 113 patients in the Control group and 221 in the PHC group were examined. The baseline demographics and intraoperative variables of patients are shown in Table 1. Participant characteristics were similar between the two groups (*P* > 0.05).

**Table 1:** Baseline demographics and intraoperative variables

Variable	Control group (*n* = 113)	PHC group (*n* = 221)	*P*-value
Age (years)	34 ± 9	33 ± 8	0.347
Sex (Male)	25 (22)	68 (31)	0.091
Height (cm)	168 ± 8	168 ± 8	0.909
Weight (kg)	108 ± 25	107 ± 23	0.503
BMI (kg/m^2^)	38 ± 7	38 ± 7	0.391
ASA physical status			
II	49 (43)	103 (47)	0.573
III	64 (57)	118 (53)	
Apfel risk score			
0	2 (2)	12 (5)	0.126
1	13 (11)	39 (18)	
2	48 (42)	97 (44)	
3	46 (41)	67 (30)	
4	4 (4)	6 (3)	
Comorbidities			
Diabetes mellitus	42 (37)	84 (38)	0.881
Hypertension	22 (20)	43 (20)	0.998
Smoking	17 (15)	46 (21)	0.196
History of motion sickness	3 (3)	6 (3)	0.974
Types of surgery			
LSG	70 (62)	134 (61)	0.904
LSG-JJB	28 (25)	55 (25)	
LSG-DJB	10 (9)	18 (8)	
OAGB	5 (4)	14 (6)	
Duration of the anesthesia (min)	93 ± 22	92 ± 23	0.619
Duration of the operation (min)	77 ± 22	76 ± 22	0.867
IOC (mg)	56.8 ± 6.9	58.2 ± 7.5	0.103
Sugammadex	34 (30)	61 (28)	0.635
Neostigmine	79 (70)	160 (72)	0.635
Fluid infusion (ml)	1369 ± 341	1402 ± 370	0.433
Fluid output (ml)	203 ± 67	191 ± 83	0.183
Duration of mechanical ventilation (min)	120 ± 28	120 ± 27	0.768
Duration of PACU stay (min)	66 ± 27	66 ± 23	0.968
Rescue opioids	75 (66)	124 (56)	0.071

Categorical data are presented as ***n*** (%), and continuous data are presented as mean ± standard deviation

***Abbreviations: ASA*** American Society of Anesthesiologists, ***BMI*** body mass index, ***IOC*** intraoperative opioids consumption (as intravenous morphine equivalent), ***LSG*** laparoscopic sleeve gastrectomy, ***LSG-DJB*** laparoscopic sleeve gastrectomy plus duodenojejunal bypass, ***LSG-JJB*** laparoscopic sleeve gastrectomy plus jejunojejunal bypass, ***OAGB*** one anastomosis gastric bypass, ***PACU*** postanesthesia care unit, ***PHC*** penehyclidine hydrochloride

**Fig. 2:**
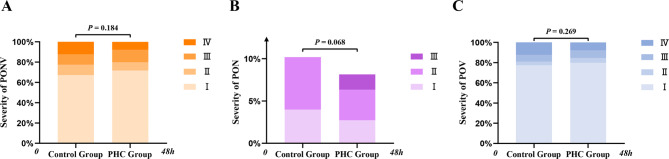
Stacked bar charts showing the severity of PONV (**A**), PON (**B**), and POV (**C**) within 48 h postoperatively between the two groups. The severity of PONV was evaluated as follows: I = no nausea or vomiting, II = nausea but no vomiting, III = mild to moderate vomiting, and IV = severe and frequent vomiting more than five times within 24 h. The severity of PON was evaluated as follows: I = mild, II = moderate, III = severe. The severity of POV was evaluated as follows: I = no vomiting, II = vomiting episodes occurring 1–2 times within 24 h, III = vomiting episodes occurring 3–5 times within 24 h, IV = vomiting episodes occurring > 5 times within 24 h. *PONV* postoperative nausea and vomiting, *PON* postoperative nausea, *POV* postoperative vomiting

PONV occurred in 159 (48%) patients within the first 48 h postoperatively, including 58 (51%) patients in the Control group and 101 (46%) in the PHC group (Table 2). There was no significant difference in the incidence or severity of PONV between the two groups (*P* > 0.05). Within the first 24 h postoperatively, 48% of the patients experienced PONV. The incidence of PONV decreased to 14% in the Control group and 11% in the PHC group within 24–48 h after surgery (Table 3). As shown in Table 3; Fig. 2, the incidence of PONV was almost the same in both groups during the two time periods (*P* > 0.05). Specifically, no significant difference was found in the incidence or severity of PONV, PON, or POV within the first 48 h after LBS (*P* > 0.05).

**Table 2:** Comparison of primary and secondary outcomes within 48 h postoperatively between the two groups

Variable	Control group (*n* = 113)	PHC group (*n* = 221)	*P*-value
PONV	58 (51)	101 (46)	0.330
PON	12 (11)	22 (10)	0.850
POV	46 (41)	79 (36)	0.377
Rescue antiemetic therapy	30 (27)	49 (22)	0.376
Water intake (ml)	1645 ± 506	1571 ± 515	0.212

Categorical data are presented as ***n*** (%), and continuous data are presented as mean ± standard deviation

***Abbreviations: PHC*** penehyclidine hydrochloride, ***PONV*** postoperative nausea and vomiting, ***PON*** postoperative nausea, ***POV*** postoperative vomiting

**Table 3:** Comparison of primary and secondary outcomes within the first 24 h and 24–48 h postoperatively between the two groups

Variable	0–24 h after surgery		*P*-value	24–48 h after surgery		*P*-value
	Control group (*n* = 113)	PHC group (*n* = 221)		Control group (*n* = 113)	PHC group (*n* = 221)	
PONV	58 (51)	101 (46)	0.330	16 (14)	24 (11)	0.385
Severity of PONV						
I	55 (49)	120 (54)	0.481	97 (86)	197 (89)	0.147
II	12 (11)	22 (10)		11 (10)	14 (6)	
III	21 (19)	45 (20)		2 (2)	9 (4)	
IV	25 (22)	34 (15)		3 (3)	1 (1)	
PON	12 (11)	22 (10)	0.850	11 (10)	14 (6)	0.273
Severity of PON						
I	5 (4)	6 (3)	0.051	4 (4)	6 (3)	0.236
II	7 (6)	10 (5)		7 (6)	6 (3)	
III	0 (0)	6 (3)		0 (0)	2 (1)	
POV	46 (41)	79 (36)	0.377	5 (4)	10 (5)	0.967
Severity of POV						
I	67 (59)	142 (64)	0.489	108 (96)	211 (96)	0.133
II	6 (5)	15 (7)		2 (2)	6 (3)	
III	15 (13)	30 (14)		0 (0)	3 (1)	
IV	25 (22)	34 (15)		3 (3)	1 (1)	
Rescue antiemetic therapy	30 (27)	49 (22)	0.376	3 (3)	5 (2)	0.826
Water intake (ml)	450 ± 160	459 ± 158	0.628	1195 ± 380	1112 ± 390	0.065

Categorical data are presented as ***n*** (%), and continuous data are presented as mean ± standard deviation

***Abbreviations: PHC*** penehyclidine hydrochloride, ***PONV*** postoperative nausea and vomiting, ***PON*** postoperative nausea, ***POV*** postoperative vomiting

As shown in Tables 2 and 3, no significant difference was observed in the proportion of patients receiving postoperative rescue antiemetic therapy or the amount of water intake between the two groups during the two postoperative periods (*P* > 0.05).

**Fig. 3:**
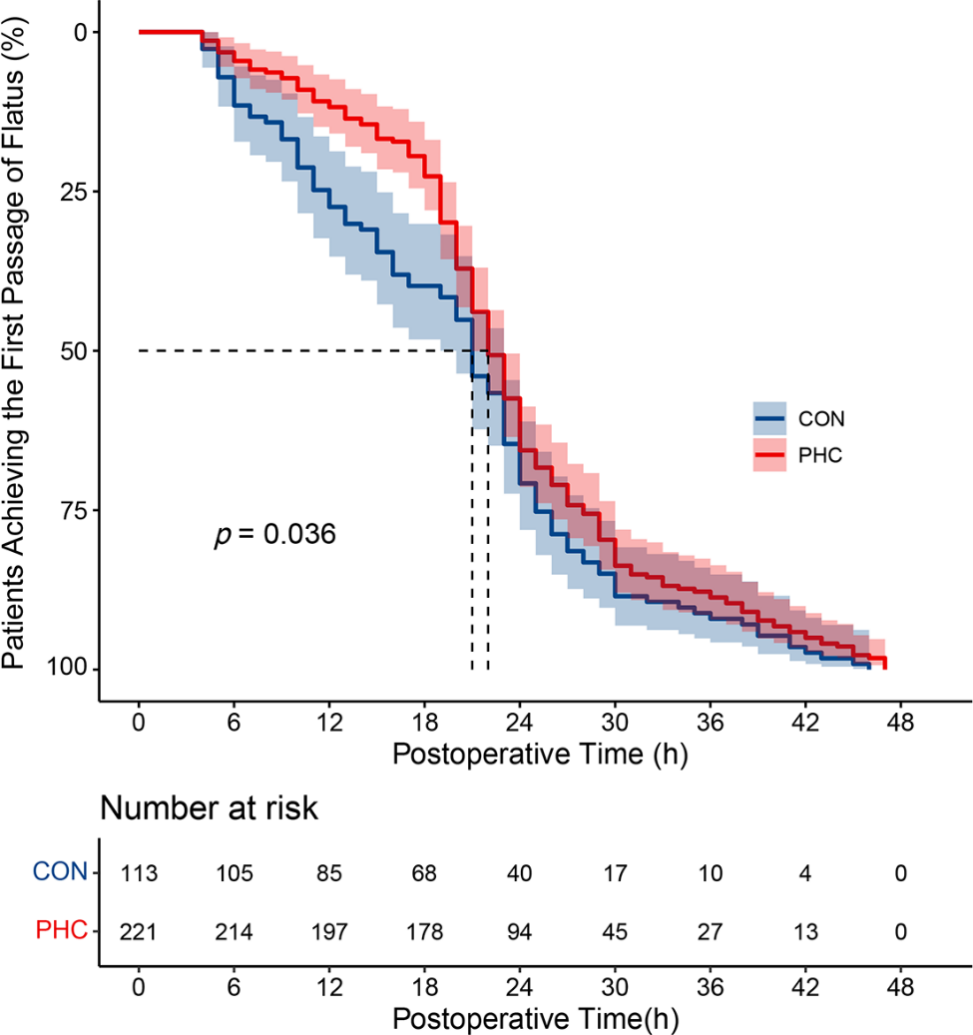
Kaplan-Meier curves of the time to first flatus postoperatively between the two groups. *CON* control; *PHC* penehyclidine hydrochloride

As shown in Fig. 3, the time to first flatus was significantly higher in patients in the PHC group (median onset time, 22 h) than in those in the Control group (median onset time, 21 h), as shown by the Kaplan–Meier curves (*P* = 0.036).

## Discussion

In this prospective, double-blinded, randomized controlled trial, our findings show that 48% of the patients experienced PONV within the first 48 h postoperatively, with 51% in the Control group and 46% in the PHC group. Moreover, the expected results were not obtained. During the early postoperative period, penehyclidine failed to induce a significant difference with respect to the incidence and severity of PONV, PON, and POV; need for rescue antiemetic therapy; or volume of water intake. However, a single intravenous dose of penehyclidine (0.5 mg) was associated with a slightly prolonged time to first flatus.

The consensus guidelines for the management of PONV include bariatric surgery as having an increased risk of PONV [17]. However, the complex etiology and pathophysiology of PONV remain elusive. The gut vagal afferent fibers innervate the gastrointestinal tract and constitute a specific neural pathway to the nucleus of the solitary tract in the hindbrain that triggers vomiting [18, 19]. Considering that surgical manipulation involves the gastric vagal nerve, the stimulation of these fibers may be responsible for the high incidence of PONV after LBS, especially in LSG. This would explain why patients undergoing LSG are more prone to develop PONV than those undergoing other bariatric procedures [3, 4]. Moreover, current evidence strongly suggests a role for the use of CO_2_ pneumoperitoneum in the pathogenesis of PONV [20, 21]. The CO_2_ pneumoperitoneum is attributed to increased intra-abdominal pressure, which decreases intestinal blood flow, especially in obese patients. In addition to residual intra-abdominal CO_2_, a recently published retrospective study by Lu et al. [22] suggested that aseptic inflammation caused by ischemia and hypoxia also plays a role in the occurrence of PONV by inducing the release of a variety of transmitters. The reason is that the intestine is probably the most sensitive internal organ for ischemia. Even short periods of ischemia can induce the release of neurotransmitters, such as serotonin, that could stimulate the emetic chemoreceptor trigger zone and result in PONV [7, 23]. Given the high levels of serotonin in the gut, exposing the gut to surgical procedures and anesthetics may increase the excitability of the gut–vagus–brain reflex that contributes to PONV [24]. Furthermore, PONV is associated with a higher gastric intraluminal pressure and less distensibility after bariatric surgery [25, 26].

To date, penehyclidine, as a preanesthetic medication, is used mainly to reduce respiratory secretions and inhibit the vagus nerve reflex without increasing the heart rate. Nevertheless, there is insufficient high-quality evidence to confirm the protective effect of penehyclidine against PONV. In a recent prospective study of 228 pediatric patients undergoing strabismus surgery, patients receiving penehyclidine (0.01 mg/kg, maximal dose 0.5 mg, intravenously) after anesthesia induction had a significantly lower incidence and severity of PONV within 48 h postoperatively [11]. The result of the marked reduction of PONV by penehyclidine administration was revealed in the pediatric and non-obese population, with an intervention performed not on gastrointestinal tract but for strabismus. However, Zhang et al. [9] found that the incidence of POV and need for antiemetic rescue were both lower in patients receiving tropisetron, a long-acting 5-hydroxytryptamine-3 antagonist, than in patients receiving penehyclidine (0.01 mg/kg, maximal dose 1.0 mg, intramuscularly) after gynecological laparoscopic surgery. In contrast, a combination of tropisetron and penehyclidine was more effective in preventing PONV than monotherapy with either tropisetron or penehyclidine. Considering the mean elimination half-life of penehyclidine from a pharmacokinetic perspective, a low-dose bolus (0.5 mg, intravenously) plus continuous infusion (a dose of 0.25 mg added to 100 ml at a rate of 2 ml/h for 48 h, intravenously) of penehyclidine was confirmed to be more effective in reducing PONV after bimaxillary orthognathic surgery [27]. We speculated that the following reasons may be responsible. First, penehyclidine may reduce the vagus nerve reflex, which may inhibit vagal afferent activation to mitigate PONV. Second, penehyclidine has been confirmed to relieve gastrointestinal smooth muscle spasms by acting on the cholinergic receptors in the glands and smooth muscles of the digestive tract, which is effective in lowering gastric intraluminal pressure after surgery. Third, a previous study suggested that penehyclidine post-conditioning could improve small intestinal mucosal injury and reduce damage to the barrier function of the small intestinal mucosa caused by limb ischemia-reperfusion [28]. Accordingly, we presume that improving intestinal microcirculation might be beneficial in decreasing PONV. However, penehyclidine was not found to be significantly associated with a lower incidence of PONV in our study. A meta-analysis confirmed that LSG is associated with an increase in the vagal tone [29]. The dosage of penehyclidine used in the study may have been inadequate for the effective inhibition of the gut vagal afferent activation. Considering that the volume of distribution of the drug in obese patients may change, the dosage needs to be adjusted to achieve the desired effect. Therefore, we also need to bring in the assessment of the pharmacokinetics of penehyclidine when we explored its effects on PONV. Moreover, gastric denervation remains difficult to avoid during LBS and may weaken penehyclidine’s action in relieving gastrointestinal smooth muscle spasms. In addition, whether penehyclidine has an effect on intra-abdominal pressure and the release of neurotransmitters warrants further investigation.

Postoperative water intake volume and time to first flatus are two important indices for evaluating recovery of gastrointestinal function after surgery. Early oral hydration after laparoscopic cholecystectomy has been associated with a lower incidence of PONV in the ward [30]. This may be owing to two possible reasons. First, patients exhibiting a low incidence of PONV are more likely to drink more water. Early oral hydration and increased water intake reduce postoperative gastrointestinal tract dysfunction and accelerate gastric emptying, which is beneficial for lowering PONV. Second, theoretically, penehyclidine use may be associated with delayed recovery of intestinal functions postoperatively owing to its anticholinergic effects on the gastrointestinal tract. Indeed, we found that penehyclidine was significantly associated with a prolonged time to the first flatus. But this difference is just one hour, which may be not clinically significant. In the present study, patients on penehyclidine treatment also showed a trend of lower postoperative water intake volume within 24–48 h after surgery, but the difference was not significant. Although the use of anticholinergic agents has been verified to be the main independent risk factor for moderate-to-severe postoperative thirst [31], this effect appeared to be modest after LBS, especially in the ward.

A few issues concerning our study need to be clarified. Sugammadex and neostigmine are both commonly used to antagonize NMBDs-induced neuromuscular paralysis in the clinical setting. Considering its beneficial effects on providing fast recovery of neuromuscular function, we tend to recommend sugammadex for the patients with BMI ≥ 35 kg/m^2^ as a NMB reversal agent in our center upon their informed consent. Therefore, the differences in the selection of NMBDs and their reversal agents may call into the question whether the use of these medication is associated with PONV. However, we detected no difference in the use of NMB reversal agent between the two groups. Furthermore, rocuronium and cis-atracurium are both intermediate-acting nondepolarizing NMBDs, which means the effect of the two drugs are comparable. Although the train of four is not routinely used for monitoring of curarization during surgery in our center, both groups strictly followed the same indications for extubation and discharge from PACU. Considering that our primary outcome is the incidence and severity of PONV during postoperative stay in the ward, it seems unlikely that the choice of NMBDs and the degree of intraoperative curarization would influence PONV long after administration in the present study.

This study has some limitations. First, we just enrolled patients undergoing elective LBS, who are aged 18–60 years and ASA physical status I–III. Futhermore, this was a single-center study with relatively small sample size, which limits the generalizability of the results. The results of this trial should be considered with caution. In the future, large multi-center randomized controlled prospective studies should be conducted to comprehensively observe the safety and efficacy of penehyclidine in patients undergoing LBS. Second, further in-depth studies are needed to determine the appropriate dosage of penehyclidine to prevent PONV in obese patients, which needs to bring in the assessment of the volume of distribution. An improved understanding of the pathophysiology of PONV in patients undergoing LBS is needed to guide future studies.

## Conclusions

Penehyclidine did not decrease PONV in patients who underwent LBS. As “metabolic surgery” is increasingly recognized by the public, further well-designed randomized controlled trials are warranted to validate the results and provide high-quality evidence for improving antiemetic treatment for the prevention and management of PONV.

## Data Availability

The datasets generated and/or analyzed during the current study are not publicly available due to potential patient privacy compromise but are available from the corresponding author on reasonable request.

## Abbreviations

ASA: American Society of Anesthesiologists
BMI: body mass index
BP: blood pressure
CONSORT: Consolidated Standards of Reporting Trials
ECG: electrocardiogram
EtCO_2_: end-tidal carbon dioxide
HR: heart rate
IOC: intraoperative opioids consumption
LBS: laparoscopic bariatric surgery
LSG-DJB: laparoscopic sleeve gastrectomy plus duodenojejunal bypass
LSG-JJB: laparoscopic sleeve gastrectomy plus jejunojejunal bypass
NMB: neuromuscular blockade
NMBDs: neuromuscular blocking drugs
OAGB: one anastomosis gastric bypass
PACU: post-anesthesia care unit
PHC: penehyclidine hydrochloride
PONV: postoperative nausea and vomiting
PON: postoperative nausea
POV: postoperative vomiting
SpO_2_: oxygen saturation
